# Capsule release surgery temporarily reduces contracture in a rat elbow model of arthrofibrosis

**DOI:** 10.1002/jor.25967

**Published:** 2024-09-15

**Authors:** Austin J. Scholp, Jordan A. Jensen, Timothy P. Fowler, Emily Petersen, Douglas Fredericks, Aliasger K. Salem, Dongrim Seol, Mitchell Coleman, Spencer P. Lake, James A. Martin, Edward A. Sander

**Affiliations:** ^1^ Roy J. Carver Department of Biomedical Engineering University of Iowa Iowa City Iowa USA; ^2^ Department of Orthopedics and Rehabilitation University of Iowa Iowa City Iowa USA; ^3^ Department of Pharmaceutical Science University of Iowa Iowa City Iowa USA; ^4^ Department of Mechanical Engineering & Materials Science Washington University in St. Louis St. Louis Missouri USA; ^5^ Department of Orthopaedic Surgery Washington University in St. Louis St. Louis Missouri USA

**Keywords:** arthrofibrosis, blebbistatin, capsule release surgery, elbow joint, rat model

## Abstract

Elbow trauma can lead to joint contracture and reduced range of motion (ROM). Nonsurgical interventions can improve ROM, but in some cases capsule release surgery is required. Although surgery can improve ROM, it often does not restore full ROM. Thus, alternatives are needed. One approach is to target activated myofibroblasts, which are commonly associated with fibrotic tissue. Mechanical and biochemical cues drive a feedback loop that can result in normal or pathological healing. We hypothesize that this feedback loop exists in joint contracture and can be manipulated so that myofibroblast activity is reduced, normal healing is achieved, and ROM is improved. We previously demonstrated that blebbistatin can inhibit myofibroblast contractile forces and reduce collagen synthesis in vitro. Thus, the purpose of this study was to assess the use of blebbistatin in an animal model of elbow contracture, which was induced in 7 groups of 4 rats each (*n* = 28). All elbows were mechanically and histologically tested. The uninjured contralateral elbows of each rat were used as a control group. Capsule release surgery significantly improved (*p* < 0.01) outcomes 1 week after surgery compared to injury alone and was not significantly different from uninjured elbows. Three weeks after surgery, outcomes worsened, indicating joint stiffening consistent with what is observed clinically. The addition of blebbistatin did not significantly improve outcomes. Future work will investigate relationships among treatment, fibrotic tissue deposition, myofibroblast activity, and biomechanics to determine if blebbistatin is a useful adjunctive therapy for treating joint contracture.

## 1 INTRODUCTION

The elbow is the second most dislocated joint with about 5 in 100,000 people 10 years of age or older experiencing an elbow dislocation each year.^1^ Up to 50% of patients with elbow dislocations experience stiffness and permanent loss of joint range of motion (ROM) after the injury.^2^ Furthermore, about 8% of patients with surgically repaired elbow fractures experience joint contracture within a year after surgery.^3^ Overall, between 10% and 15% of elbow fractures and dislocations lead to severe joint contractures that require surgical treatment.^4^, ^5^ Adjacent joints compensate poorly for loss of elbow ROM, resulting in functional disability of the upper limb, which limits a patient's ability to perform activities of daily living (e.g., tying shoes, brushing teeth, drinking from a cup).^6^

Current treatment strategies focus on prevention of capsular contracture by beginning physical therapy of the elbow joint as soon as possible after injury. Despite early mobilization, contracture of the elbow is still common.^7^, ^8^ If contracture is severe enough and does not respond to nonsurgical intervention, then surgical capsular release may be performed.^9^ Capsule release surgeries generally improve but do not normalize elbow ROM.^5^, ^10^, ^11^, ^12^ Charalambous and Morrey found in 9 out of 21 clinical outcome studies that open release surgery failed to improve average patient postoperative ROM to a functional range.^13^ Thus, new alternative or adjunctive treatments are needed urgently to improve patient outcomes, either through prevention or correction of established contractures.^13^, ^14^

The loss of ROM seen in joint contractures is often attributed to changes in cellular activity within the joint capsule that render it abnormally thick and stiff.^15^ Several studies have observed a significant increase of activated myofibroblasts in the contracted joint capsule of both human elbows^16^, ^17^, ^18^ and rabbit knees.^19^, ^20^, ^21^ Results suggest that myofibroblasts are a driving force behind posttraumatic joint contracture.

Myofibroblasts in a healing wound exert contractile forces on the extracellular matrix (ECM) and synthesize collagen and other ECM proteins in a manner that is modulated in part by both mechanical forces and biochemical factors (e.g., transforming growth factor (TGF)‐β1).^22^ These components interact dynamically and reciprocally with myofibroblasts, such that the wound healing response proceeds in a feedback loop that results in either a normal or a pathological healing outcome.^23^ We hypothesize that this feedback loop is operational in the injured joint capsule, where the combination of mechanical and biochemical cues ultimately leads to capsule thickening, stiffening, and contracture. Hildebrand has postulated a similar mechanism underpinning contracture in the elbow referred to as the myofibroblast–mast cell–neuropeptide axis of fibrosis.^24^ Central to both hypotheses is the idea that multiple stimuli amongst multiple cell phenotypes interact in a complex manner, such that overactive myofibroblasts are triggered by mechano‐chemical cues to produce fibrotic tissue. Therefore, strategies that can modulate components of this feedback loop could be useful targets to prevent contracture and encourage normal healing.

One such treatment target is the force sensing/generating machinery of the myofibroblast.^25^ Non‐muscle myosin II (NMMII) is a critical component of the cytoskeleton implicated in tissue fibrosis.^26^, ^27^ Phosphorylated NMMII engages with actin microfilaments to generate force through the anchoring junctions (i.e., focal adhesions) with the ECM.^28^, ^29^ These contractile forces trigger the release and activation of ECM‐bound TGF‐β1, which can further upregulate alpha smooth muscle actin (α‐SMA) expression, contractile forces, and collagen synthesis, such that fibrotic tissue is formed. Blebbistatin is a membrane permeable small molecule that quickly and reversibly inhibits NMMII in a dose‐dependent manner.^30^, ^31^ Myofibroblasts exposed to blebbistatin exhibit diminished mechanosensitivity and stimulation from TGF‐β1, and thus respond by reducing both traction forces and collagen production until the drug wears off.

Previously, we demonstrated that blebbistatin significantly reduced fibroblast force development and collagen production in vitro.^32^, ^33^ The use of this drug in conjunction with capsule release surgery might offer a way to maintain ROM gains from release surgery that are generally lost during convalescence. To test this idea, we adopted a validated Long‐Evans rat model of elbow contracture developed by Lake et al.^34^, ^35^, ^36^ We then adapted the model to investigate the effects of capsule release surgery on improving ROM and the impact of adjunctive treatment with blebbistatin.

## 2 MATERIALS & METHODS

### 2.1 Animal study design

This protocol (#2072222) was approved by the University of Iowa Institutional Animal Care and Use Committee (IACUC). All animals were housed at the University of Iowa Animal Research Surgicenter in 30.8 × 30.8 × 18.72 cm cages with a floor area of 742.2 cm^2^. Two rats were housed per cage and were fed 7913‐Irradiated NIH‐31 Modified 6% Mouse and Rat Diet. Rats were paired according to their euthanasia date and were housed in the same cages throughout the duration of this study. Evidence of previous joint‐related pathology (e.g., healed periarticular fracture) was grounds for exclusion from this study.

Long‐Evans rats were selected for this study due to their anatomical and physiological similarities with the human elbow.^37^, ^38^, ^39^ Joint contracture was induced in *n* = 28 male rats between 12 and 15 weeks of age (average weight 0.472 ± 0.037 kg,) via the surgical injury described in Lake et al.^34^ and in the next section. During a bandage change, one rat died unexpectedly and inexplicably 3 weeks after injury leaving the number of rats tested at *n* = 27. Consequently, rats were randomly split into 1 group of 3 and 6 groups of 4. No other rats were excluded from this study.

**Figure 1:**
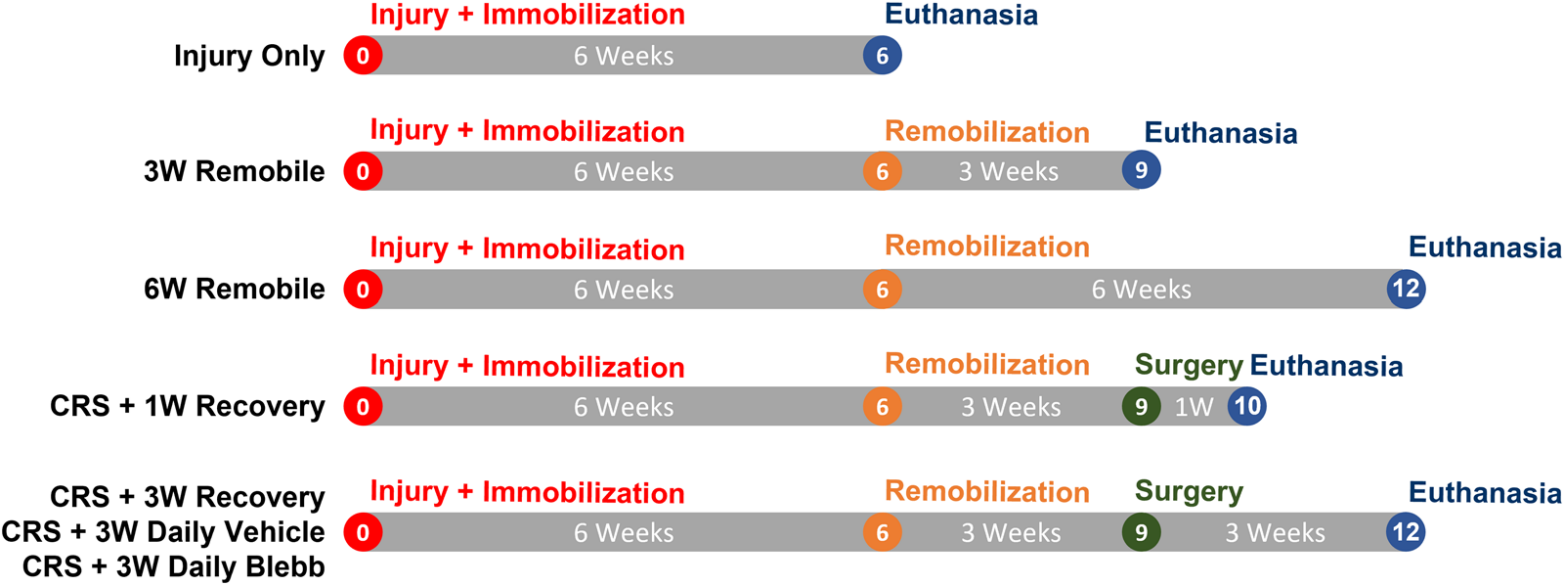
Timelines for the seven treatment groups. Contracture was induced in all groups via surgical injury followed by 6 weeks with the injured arm immobilized. Groups other than the Injury Only group had restraints removed (remobilization) after the 6‐week immobilization period and were handled as indicated. CRS – capsule release surgery.

Seven treatment groups were investigated following the timeline outlined in Figure 1. After contracture was established (6 weeks post‐injury) the first group of 3 rats was euthanized. This group provided a reference for untreated injury (referred to as *Injury Only*). The remaining 6 groups had restraints removed (i.e., remobilization) and were allowed to freely use their injured arms for either 3 or 6 weeks in the same environment in which they were immobilized. The second and third groups were euthanized after 3 and 6 weeks of remobilization (referred to as *3W Remobile* and *6W Remobile*, respectively) and provided a reference for natural recovery. The fourth and fifth groups received capsule release surgery (CRS) after 3 weeks of remobilization, followed by either 1 or 3 weeks of recovery (*CRS* + *1W Recovery* and *CRS* + *3W Recovery*, respectively). After 3 weeks of remobilization, the sixth and seventh groups received capsule release surgery and treatment with either 3 weeks of daily injections of blebbistatin (*CRS* + *3W Daily Blebb*) or daily vehicle (*CRS* + *3W Daily Vehicle*), as indicated in Figure 1. The vehicle was 0.9% saline with 6.7% dimethyl sulfoxide (DMSO) and 25% hydroxypropyl β‐cyclodextrin (w/v), a complexing agent that improves blebbistatin solubility. The contralateral uninjured arms of all rats were used as a normal uninjured control group.

### 2.2 Injury model

**Figure 2:**
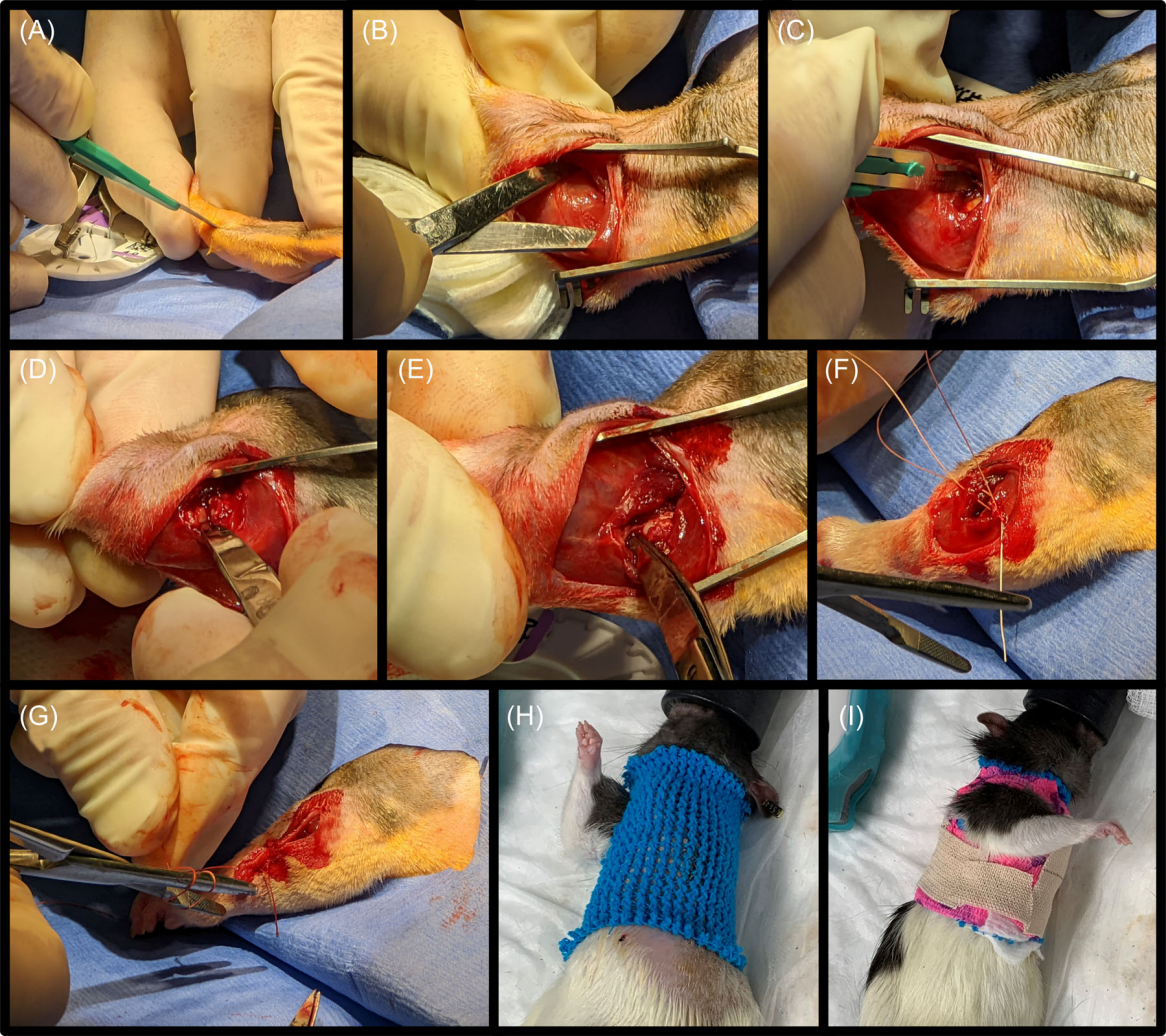
(A) An incision was made on the lateral aspect of the left elbow. (B) Blunt dissection exposed the lateral aspect of the triceps. (C, D) The anterior muscles of the brachium were lifted; capsulotomy and transection of the lateral collateral ligament were performed. (E) The arm was extended to subluxate the joint. (F) Vicryl sutures were used to close the surgical site. (G) A second layer of sutures closed the skin. (H) Tubular elastic netting was placed around the upper torso with a hole cut to allow unrestricted use of the right limb. (I) Vetrap was wrapped around the torso, securing the injured arm in place while allowing full motion of the uninjured arm.

All surgical procedures were performed at the University of Iowa Animal Research Surgicenter. Anesthesia was induced using 0.5%–3% isoflurane in oxygen. Antibiotic prophylaxis consisted of a single subcutaneous dose of enrofloxacin at 5 mg/kg and a single dose of extended‐release buprenorphine at 1 mg/kg was given for analgesia. A 2 cm incision was then made on the lateral aspect of the left elbow centered at the lateral epicondyle. Blunt dissection exposed the lateral aspect of the triceps and the common extensor origin at the lateral epicondyle. The anterior compartment muscles of the brachium were lifted allowing anterior capsulotomy followed by transection of the lateral collateral ligament. The forearm was then supinated, and the elbow extended, subluxating the elbow joint. The joint was immediately reduced with the lateral collateral ligament left unrepaired. Wound closure was performed in two layers, the deep fascial and skin, with absorbable sutures (Vicryl). Immediately following surgery, the injured arm was immobilized against the body with the elbow in approximately 135 degrees of flexion. A piece of tubular elastic netting was cut and placed around the upper torso of each animal with an access hole cut in the netting to allow unrestricted use of the uninjured right limb. A piece of self‐adhering wrap (Vetrap, 3M Maplewood, MN) was then wrapped around the same portion of the torso three times with access holes cut to leave the right limb unconstrained. This procedure is illustrated in Figure 2. The injured arm was kept immobile for 6 weeks to create contracture. Postoperative pain was reduced via 2 mg/kg subcutaneous doses of meloxicam 24 and 48 h after surgery.

### 2.3 Capsule release surgery

**Figure 3:**
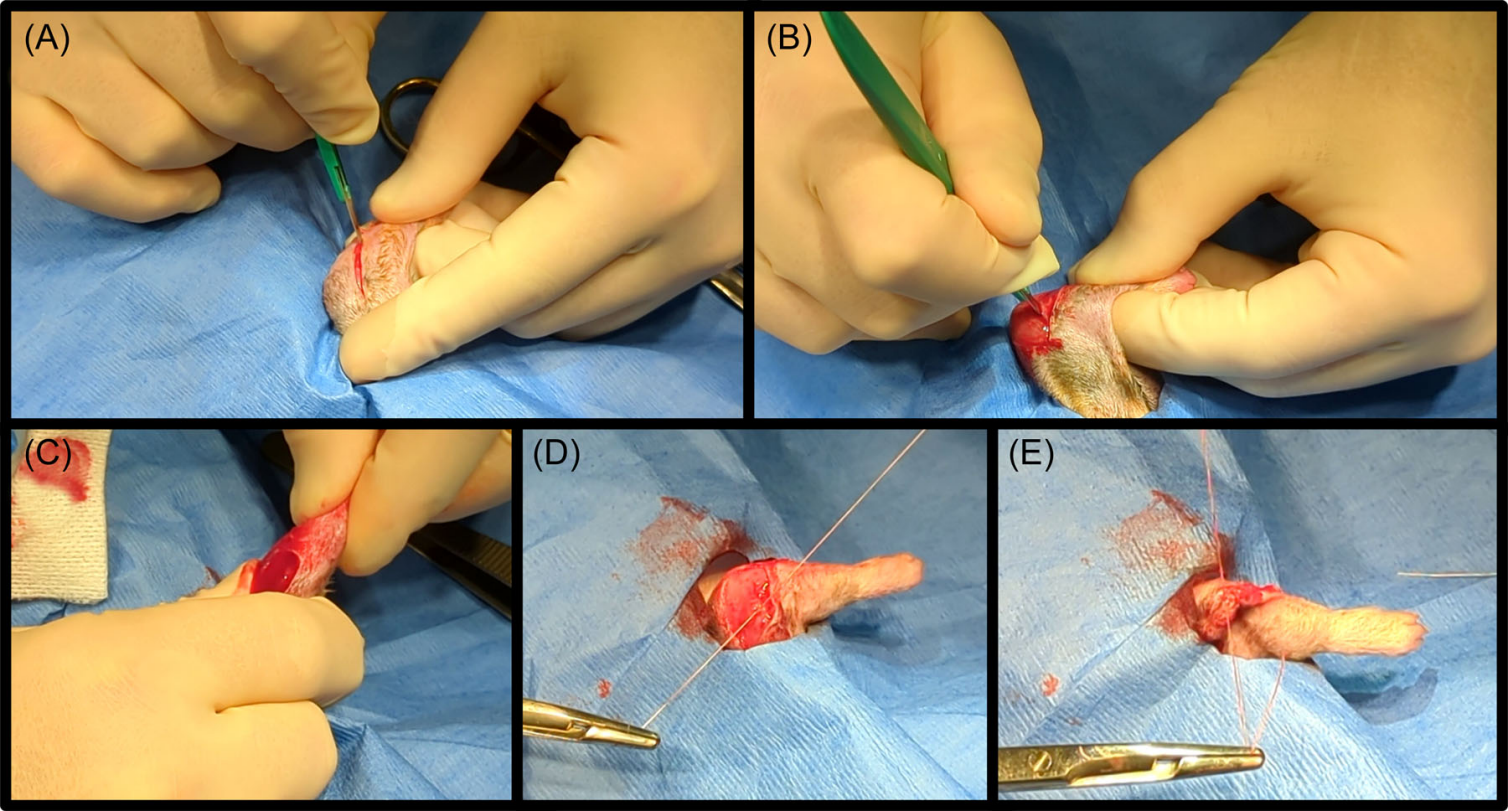
(A) An incision was made in the skin of the lateral elbow. (B) The lateral border of the humerus was identified, and the anterior capsule was released subperiosteally from the anterior cortex of the humerus. (C) The elbow was fully extended. (D, E) Absorbable sutures were used to close the wound.

Capsule release surgery was performed after 3 weeks of remobilization (Figure 3). Anesthesia was induced using 0.5‐3% isoflurane in oxygen. Antibiotic prophylaxis again consisted of a single subcutaneous dose of enrofloxacin at 5 mg/kg and a single dose of extended‐release buprenorphine at 1 mg/kg was given for analgesia. A 2 cm skin incision was made over the lateral elbow utilizing the previously made incision. The lateral border of the humerus was identified, and the muscles of anterior compartment of the brachium were elevated off the anterior cortex of the humerus. Proceeding in a proximal to distal direction (towards the elbow joint) the anterior joint capsule was eventually encountered and elevated from the anterior cortex and articular surface of the distal humerus. The lateral collateral ligament was not transected or released. No elevation of the triceps or posterior joint capsule release was performed. The elbow was then gently extended until full extension was achieved. Closure of the deep fascial and skin layers was again performed with absorbable sutures.

### 2.4 Blebbistatin

Blebbistatin (ab120425 Abcam) was diluted to 1 mg/mL in a vehicle of 0.9% saline with 6.7% dimethyl sulfoxide (DMSO) and 25% hydroxypropyl β‐cyclodextrin (w/v). Rats received drug via systemic daily injections, delivered in a single injection at a dosage of 5 mg/kg. For the first 4 days postrelease, rats received injections intraperitoneally to ensure that peak drug concentrations were reached quickly during the early phases of healing.^40^ Subcutaneous injections were performed thereafter both to reduce handling of the forelimbs during injection, which could aggravate the healing elbow, and to lessen the potential for internal injury or injection into organs.

### 2.5 Mechanical measurement

**Figure 4:**
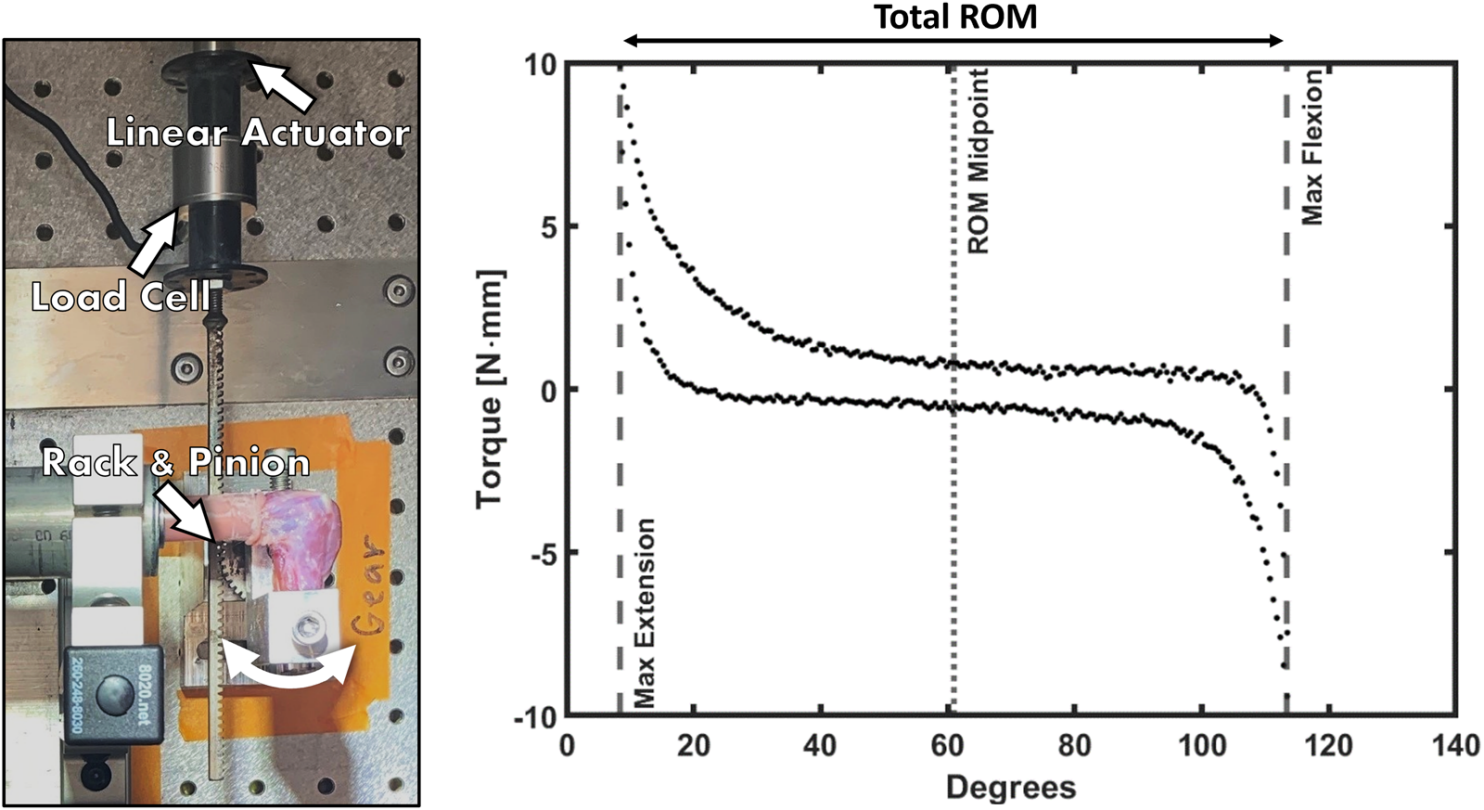
A custom‐built flexion‐extension testing system (left) was used to obtain a torque‐angle curve (right). Several biomechanical measurements can be extracted from a curve, including range of motion (ROM) and maximum extension.

Following euthanasia, the arms of all rats underwent mechanical testing similar to Lake et al.^34^ Briefly, the arm was dissected carefully to minimize disruption of any fibrotic tissue in and around the joint. The humeral head was exposed, and the paw removed at the wrist. The humeral head and wrist were mounted into polycarbonate plastic tubing and the wrist was secured to the tube with self‐curing acrylic cement (Duz‐All, Keystone Industries). The humoral head was secured with cyanoacrylate adhesive and braced with 3/8‐inch diameter, 4.5 oz., orthodontic rubber bands (IVORIE®). The arm was then mounted into a custom‐built mechanical testing system. The wrist tube was fixed in place while the humeral end was mounted into a movable lever arm (Figure 4). The testing system was controlled with a Bose ElectroForce Planar Biaxial TestBench Instrument (TA Instruments) that interfaced with a custom‐built rack and pinion system that moved the elbow through a load‐controlled cyclical motion of flexion and extension from ± 10 N·mm torque. The total ROM and maximum extension angle that each arm reached during testing were used as the primary outcome measurements.

### 2.6 Histology

After mechanical testing was completed, each arm was fixed in 10% neutral buffered formalin. Samples were decalcified in 5% formic acid and processed on an automated processor overnight. The samples were then embedded in paraffin and cut on a microtome. Sections 5 μm thick were obtained at a depth in the specimen where the capitulum of the humerus, the radial head, and part of the anterior capsule were visible. Each section was picked up on a charged slide. Following deparaffinization in xylene, slides were either stained using hematoxylin and eosin (H&E) or processed on a Discovery Ultra using 3,3′‐Diaminobenzidine (DAB) and an α‐SMA mouse monoclonal antibody (CMC20229060, Cell Marque™, Millipore Sigma) coupled with horseradish peroxidase (Discovery OmniMap Anti Mouse HRP 760‐4310, Roche Diagnostic). Immunostained sections were then counterstained with hematoxylin. Slides were imaged using an Olympus slide scanner and examined for differences in synovial fibrosis and the number of α‐SMA‐positive cells.

Peri‐articular tissues found along the capitulum between the radial and olecranon fossae were targeted for analysis of synovial fibrosis. This region was chosen as it consistently had intact synovial lining and adipose tissue that, upon histological examination, appeared minimally disturbed, if at all, by the surgical procedures. Fibrosis of this tissue was determined by the degree that dense connective tissue infiltrated into white adipose tissue,^41^ assessed via image analysis using ImageJ 1.49p (NIH). For each sample, a region of interest (ROI) was drawn on the peri‐articular tissue where both the synovial lining and adipose tissue were visible. The ROIs were then converted to 8‐bit grayscale images. Thresholding was applied to obtain black and white images, where white pixels represent connective tissue. The number of white pixels divided by the total number of pixels was used to obtain connective tissue density for each sample (Figure S1). Connective tissue density was used as a metric for changes in synovial fibrosis.

The fibrous tissue on the anterior portion of the joint was targeted for α‐SMA analysis, as α‐SMA is commonly used to identify myofibroblasts.^22^, ^42^ Images of this tissue at 5x magnification were collected from the full scan of each immunostained sample. Bone, cartilage, and muscle tissues were cropped out of each image. The number of α‐SMA positive and negative cells were then counted using the AI software platform, Biodock (Biodock 9, AI Software Platform. Biodock 2023). Briefly, a model was trained by manually selecting positive and negative cells on 5–7 subsections of three example images (Figure S2). The first version of the model classified cells in adjacent subsections of each image. Cell classifications were then manually corrected. The model was retrained using the corrected subsections. This process was repeated until no additional corrections were needed, which occurred after six iterations. All images were then analyzed with this trained model and the percentage of α‐SMA positive cells was used as the outcome measure of each sample.

### 2.7 Statistical analysis

In each treatment group, the mean, standard deviation, coefficient of variation (standard deviation divided by mean), and 95% confidence intervals were calculated for ROM, maximum extension, connective tissue density, and the percentage of α‐SMA positive cells. One‐way ANOVA was used to determine if there was a significant relationship between groups for each outcome variable. Tukey's test was used to find significant differences between individual groups. Linear regression analysis was used to determine correlations between each outcome measure. Statistical analysis was performed in Prism GraphPad 10.0.2 (GraphPad Software). A *p*‐value less than 0.05 was considered to be significant.

## 3 RESULTS

### 3.1 Remobilization after injury improves but does not normalize elbow range of motion

**Figure 5:**
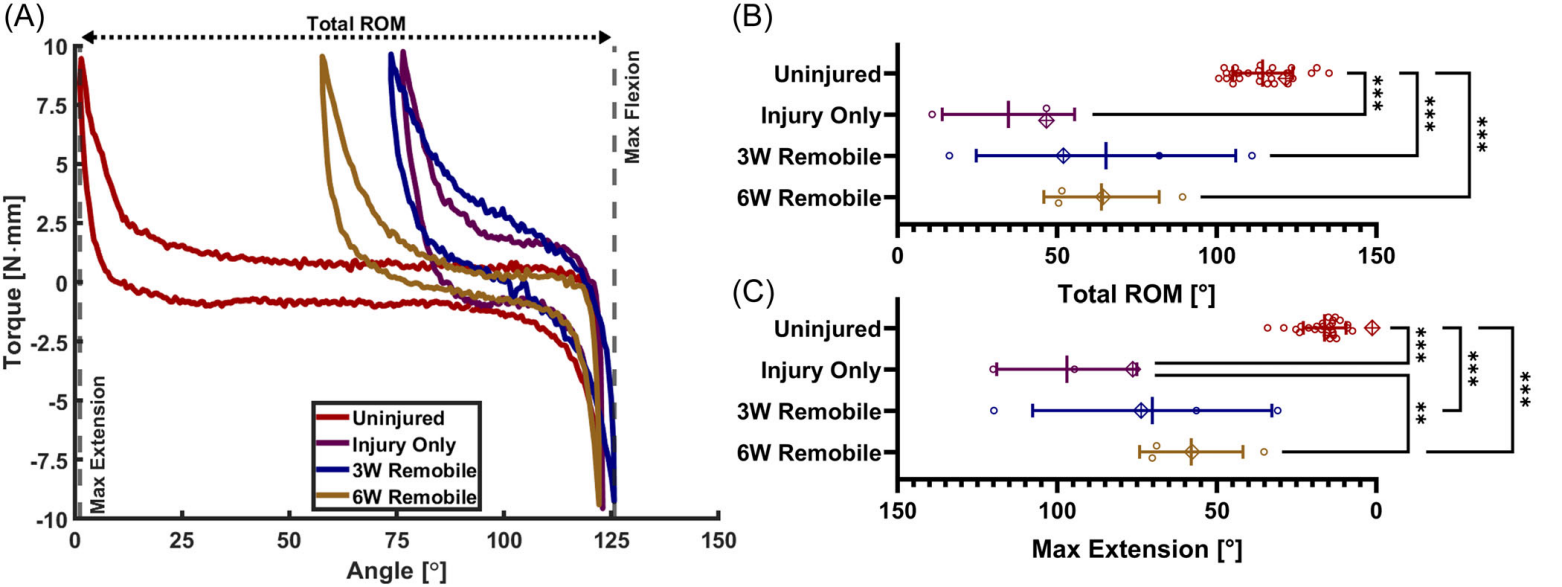
(A) Selected torque‐angle curves for the *Uninjured*, *Injury Only*, *3W Remobile*, and *6W Remobile* groups. The range of motion (ROM) (B) and max extension angles (C) extracted from the torque‐angle data. Lines denote the mean and error bars indicate the standard deviation for each group. Data points marked with a crossed diamond denote the data that correspond to the representative torque‐angle plots in (A). **p < 0.01, ***p < 0.001.

Representative torque‐angle curves (Figure 5A) clearly indicate significant reductions in ROM and maximum extension due to injury and immobilization, followed by some recovery after remobilization. The total ROM (Figure 5B) and maximum extension angles (Figure 5C, Table 1 & Supporting Information S1: Table S1) for uninjured elbows were 114 ± 9.4° and 16 ± 6.7°, respectively. Both ROM and maximum extension significantly worsened (*p* < 0.001) with injury and 6 weeks of immobilization (ROM: 35 ± 21°, Max Extension: 97 ± 22°). These ROM values were comparable to those reported by Lake et al. for uninjured (107.3 ± 10.2°) and injured (50 ± 10.6°) Long Evans rat elbows.^34^ ROM and extension improved (but were highly variable) after 3 weeks with free use of the injured arm (ROM: 65 ± 41°, Max Extension: 70 ± 37°). An additional 3 weeks (6 weeks total) of remobilization reduced variability but did not on average improve elbow ROM (64 ± 18°); however, maximum extension (58 ± 16°) did significantly (*p* < 0.01) improve compared to the injury group.

**Table 1:** Descriptive statistics for each outcome variable and each treatment group. SD–standard deviation, CoV–coefficient of variation, CI–95% confidence interval.

Group	Parameter	Mean ± SD	CoV	CI
Uninjured	ROM [degrees]	114 ± 9.4	8.20%	[111, 118]
	Max extension [degrees]	16 ± 6.7	42%	[14, 19]
	Percent α‐SMA [%]	4.98 ± 7.4	149%	[1.7, 8.26]
	Connective tissue density [%]	45 ± 6.3	14%	[41, 48]
Injury Only	ROM [degrees]	35 ± 21	60%	[−17, 86]
	Max extension [degrees]	97 ± 22	23%	[42, 151]
	Percent α‐SMA [%]	29.6 ± 50.3	170%	[−95.3, 154]
	Connective tissue density [%]	70 ± 1.5	2.2%	[66, 73]
3W Remobile	ROM [degrees]	65 ± 41	62%	[0.64, 130]
	Max extension [degrees]	70 ± 37	53%	[11, 130]
	Percent α‐SMA [%]	34 ± 31.2	91.7%	[−15.6. 83.7]
	Connective tissue density [%]	60 ± 6.5	11%	[49, 70]
6W Remobile	ROM [degrees]	64 ± 18	28%	[35, 93]
	Max extension [degrees]	58 ± 16	28%	[32, 84]
	Percent α‐SMA [%]	0.58 ± 0.67	116%	[−0.49, 1.65]
	Connective tissue density [%]	47 ± 1.7	3.7%	[44, 50]
CRS + 1W Recovery	ROM [degrees]	90 ± 7.6	8.50%	[78, 102]
	Max extension [degrees]	34 ± 6.2	18%	[24, 44]
	Percent α‐SMA [%]	15.2 ± 12.5	82.3%	[−4.7, 35.1]
	Connective tissue density [%]	60 ± 6.9	11%	[49, 71]
CRS + 3W Recovery	ROM [degrees]	81 ± 8.2	10%	[68, 94]
	Max extension [degrees]	51 ± 16	31%	[26, 77]
	Percent α‐SMA [%]	4.47 ± 5.35	120%	[−4.05, 13]
	Connective tissue density [%]	68 ± 6.7	9.9%	[57, 78]
CRS + 3W Daily Vehicle	ROM [degrees]	84 ± 3.6	4.30%	[78, 90]
	Max extension [degrees]	43 ± 5.5	13%	[34, 52]
	Percent α‐SMA [%]	0.31 ± 0.2	64.3%	[−0.01, 0.63]
	Connective tissue density [%]	70 ± 6.9	9.8%	[59, 81]
CRS + 3W Daily Blebb	ROM [degrees]	79 ± 9.1	12%	[65, 94]
	Max extension [degrees]	41 ± 9.6	23%	[26, 56]
	Percent α‐SMA [%]	0.74 ± 0.59	79.5%	[−0.2, 1.68]
	Connective tissue density [%]	61 ± 6.2	10%	[51, 71]

Abbreviations: CI, 95% confidence interval; CoV, coefficient of variation; SD, standard deviation.

### 3.2 Capsule release surgery improves elbow extension but some initial gains are lost after recovery

**Figure 6:**
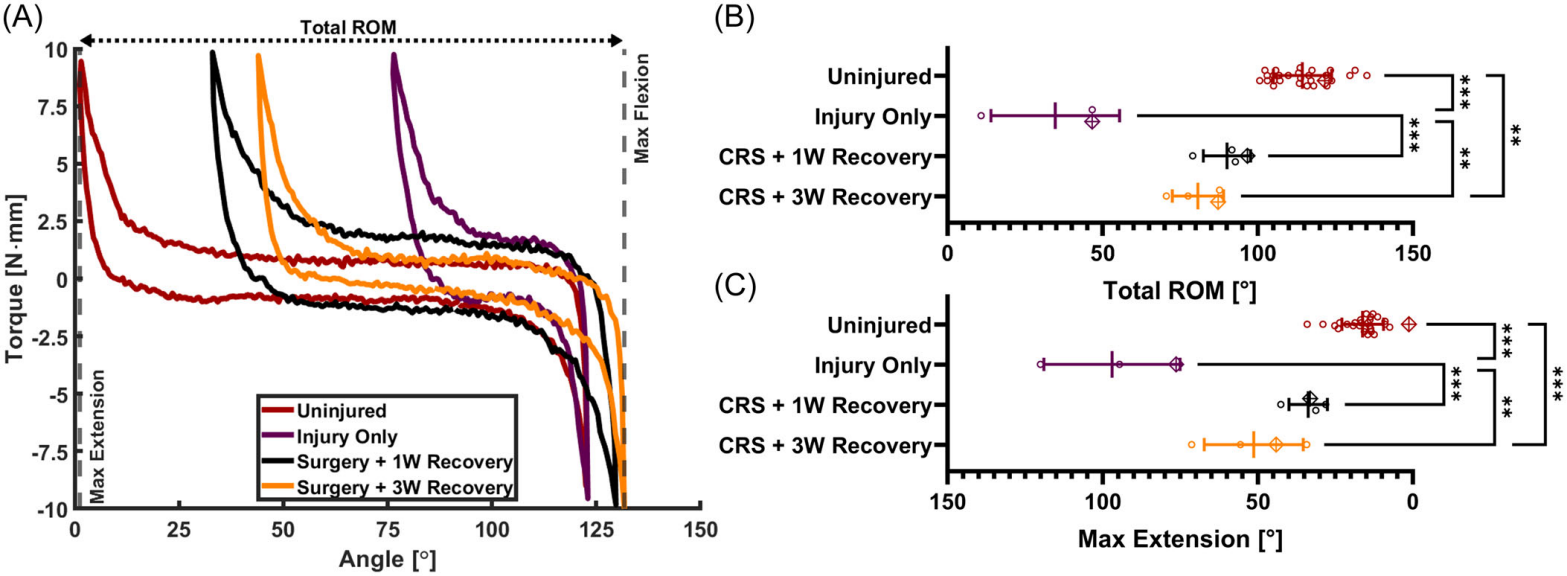
(A) Selected torque‐angle curves for the *Uninjured*, *Injury Only, CRS* + *1W Recovery*, and *CRS* + *3W Recovery* groups. The ROM (B) and max extension angles (C) extracted from the torque‐angle data. Lines denote the mean and error bars indicate the standard deviation for each group. Data points marked with a crossed diamond denote the data that correspond to the representative torque‐angle plots in (A). **p < 0.01, ***p < 0.001. CRS, capsule release surgery; ROM, range of motion.

Compared to the injury‐only group, capsule release surgery (Figure 6A) significantly improved (*p* < 0.001) ROM and maximum extension both 1 week (ROM: 90 ± 7.6°, Max Extension: 34 ± 6.2°) and 3 weeks after surgery (ROM: 81 ± 8.2°, Max Extension: 51 ± 16°). Neither ROM nor maximum extension 1 week after surgery were significantly different from the uninjured group (Figure 6B,C, Table 1 and Supporting Information S1: Table S1). Three weeks after surgery, ROM and maximum extension worsened, though not significantly, compared to 1 week. This regression in joint biomechanics reflects the loss of ROM after capsule release surgery often observed clinically.^43^

### 3.3 Blebbistatin injections do not provide mechanical benefits

**Figure 7:**
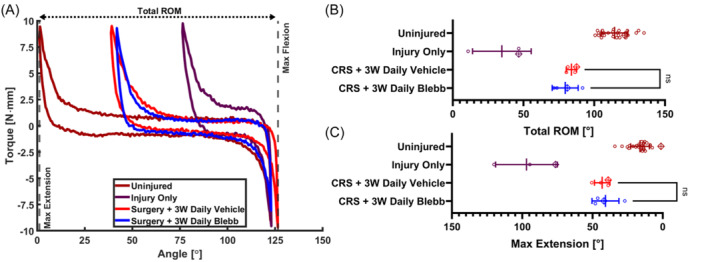
(A) Selected torque‐angle curves for the *Uninjured*, *Injury Only*, *CRS* + *3W Daily Vehicle*, and *CRS* + *3W Daily Blebb* groups. The ROM (B) and max extension angles (C) extracted from the torque‐angle data. Lines denote the mean and error bars indicate the standard deviation for each group. Data points marked with a crossed diamond denote the data that correspond to the representative torque‐angle plots in (A). Only nonsignificant relationships are shown for clarity. CRS, capsule release surgery; ROM, range of motion.

Torque‐angle curves (Figure 7A) indicate no differences between vehicle and blebbistatin injections. Total ROM and maximum extension angles (Figure 7B,C, Table 1 & Supporting Information S1: Table S1) between the groups that received daily blebbistatin (ROM: 79 ± 9.1°, Max Extension: 41 ± 9.6°) and daily vehicle injections (ROM: 84 ± 3.6°, Max Extension: 43 ± 5.5°) were not significantly different from each other or from capsule release surgery alone, indicating that blebbistatin had no benefit for improving joint biomechanics.

### 3.4 Histological analysis of the synovial lining reveals differences in connective tissue density

**Figure 8:**
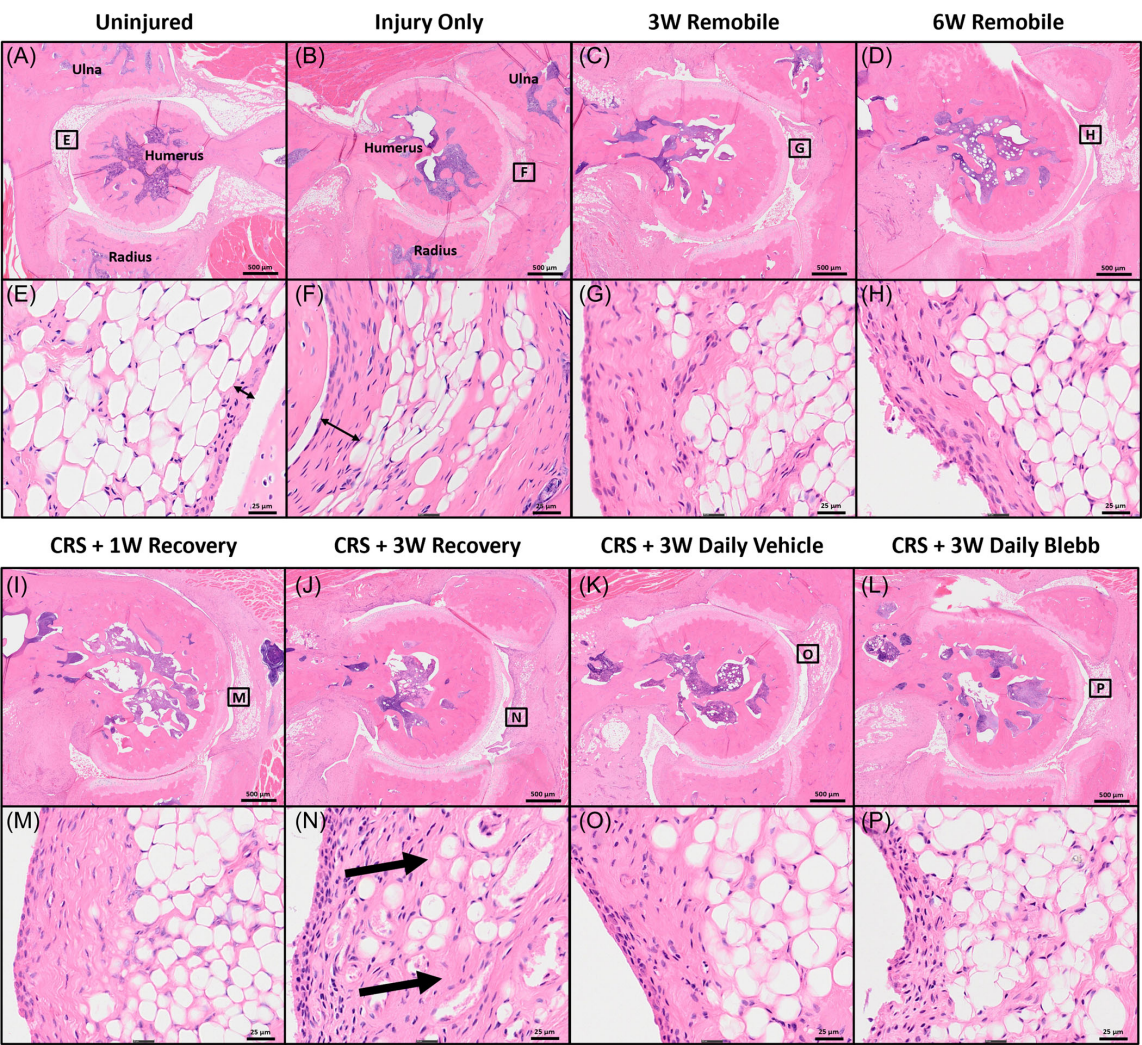
Representative images of hematoxylin and eosin stained sections for the following groups: *Uninjured* (A, E), *Injury Only* (B, F), *3W Remobile* (C, G), *6W Remobile* (D, H), *CRS* + *1W Recovery* (I, M), *CRS* + *3W Recovery* (J, N), *CRS* + *3W Daily Vehicle* (K, O), and *CRS* + *3W Daily Blebb* (L, P). Peri‐articular tissues that contain white fat are marked by rectangles with labels that correspond to the higher magnification images of each sample. Thin double‐sided arrows highlight differences in synovial thickness. Thick arrows highlight infiltration of the dense connective tissue in the white fat. CRS, capsule release surgery.

Compared to the uninjured controls, increased capsular fibrosis, synovial lining thickness, and infiltration of dense connective tissue into white adipose tissue were observed in the H&E staining of all groups (Figure 8). The synovial lining of the *CRS* + *3W Daily Blebb* group (Figure 8L,P) shows some thickening, but not to the degree seen in the other injured groups.

**Figure 9:**
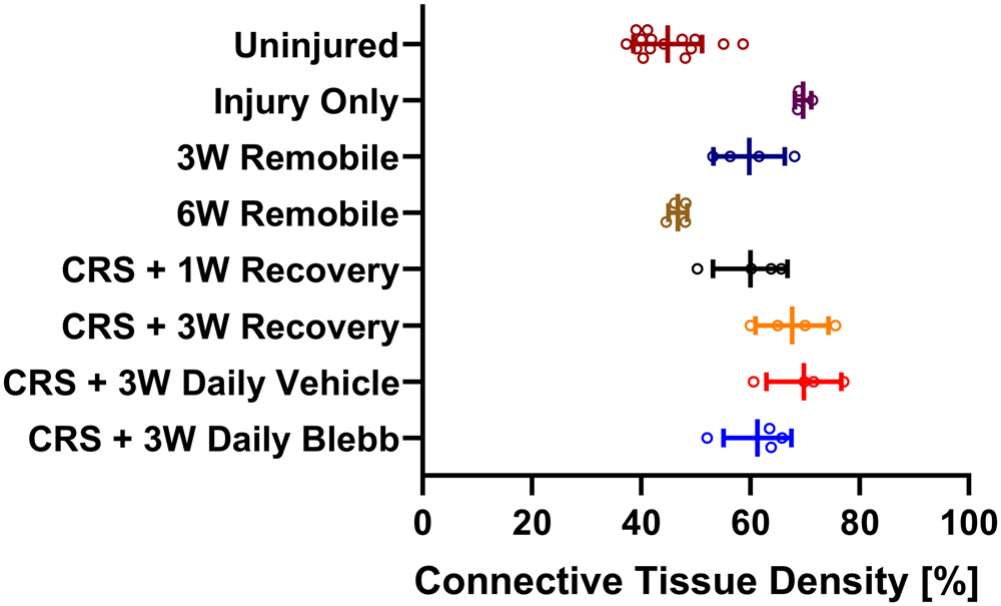
The connective tissue density of each group. Lines denote the mean and error bars indicate the standard deviation for each group. Refer to Supporting Information S1: Table S2 for significant relationships, which are not shown here for clarity. CRS, capsule release surgery.

The connective tissue density (Figure 9, Table 1 & Supporting Information S1: Table S2) for all groups other than *6W Remobile* was significantly greater (*p* < 0.01) compared to the uninjured group. Compared to injury alone, the *6W Remobile* group was the only treatment group to have significantly reduced connective tissue density (*p* < 0.001). Three weeks of remobilization (*3W Remobile*) led to a reduction in connective tissue density, but this was not a significant improvement compared to injury alone. One week after capsule release surgery (*CRS* + *1W Recovery*), elbows had lower connective tissue density than the injury‐only group, but connective tissues returned 3 weeks after surgery (*CRS* + *3W Recovery*). Neither the *CRS* + *1W Recovery* nor the *CRS* + *3W Recovery* groups were significantly different from injury alone, or from each other. The addition of blebbistatin injections mitigated the return of connective tissue, ending with similar densities to those seen 1 week after capsule release and after 3 weeks of remobilization.

### 3.5 The percentage of α‐SMA‐positive cells may not be linked to arthrofibrosis

**Figure 10:**
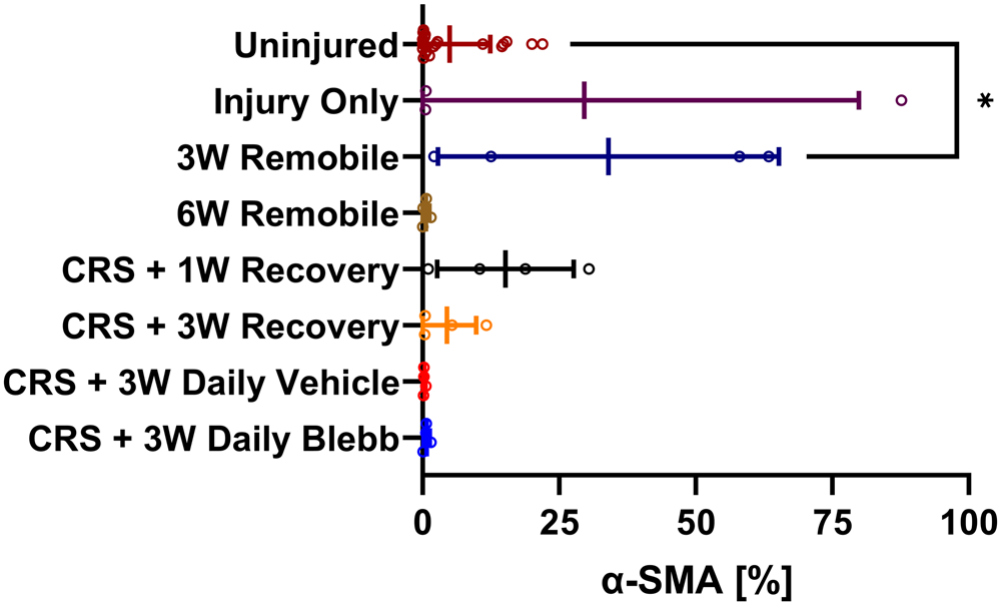
The percentage of α‐SMA‐positive cells in each treatment group. Lines denote the mean and error bars indicate the standard deviation for each group. Since negative percentages are not possible, the x‐axis is bound from 0 to 100, cutting off the error bars for the *Uninjured*, *Injury Only*, and *CRS* + *3W Recovery* groups. *p < 0.05. CRS, capsule release surgery.

**Figure 11:**
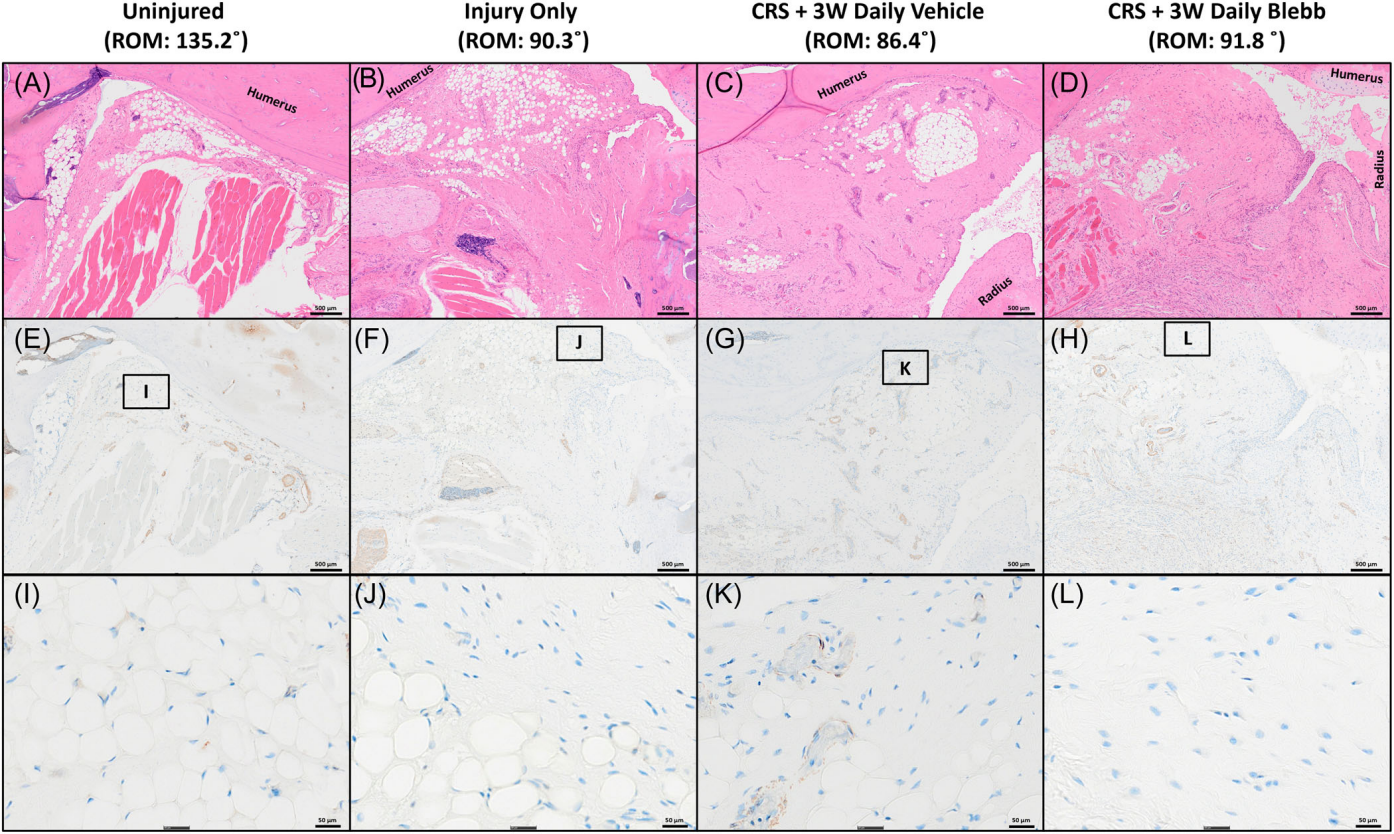
H&E stained (A–D) sections of different groups along with immunolabeled sections in the same location (E–H). Panels I–L show the magnified regions of the immunolabeled sections. Different treatments did not exhibit differences in α‐SMA expression. CRS, capsule release surgery.

The percentage of α‐SMA‐positive cells (Figure 10) in the *3W Remobile* group was significantly higher than uninjured controls (*p* < 0.05), which was the only significant difference observed among the treatment groups. The majority of injured elbows (23/27; 85.2%) had percentages lower than 22%, which was the highest percentage found in uninjured arms (Figure 11).

**Figure 12:**
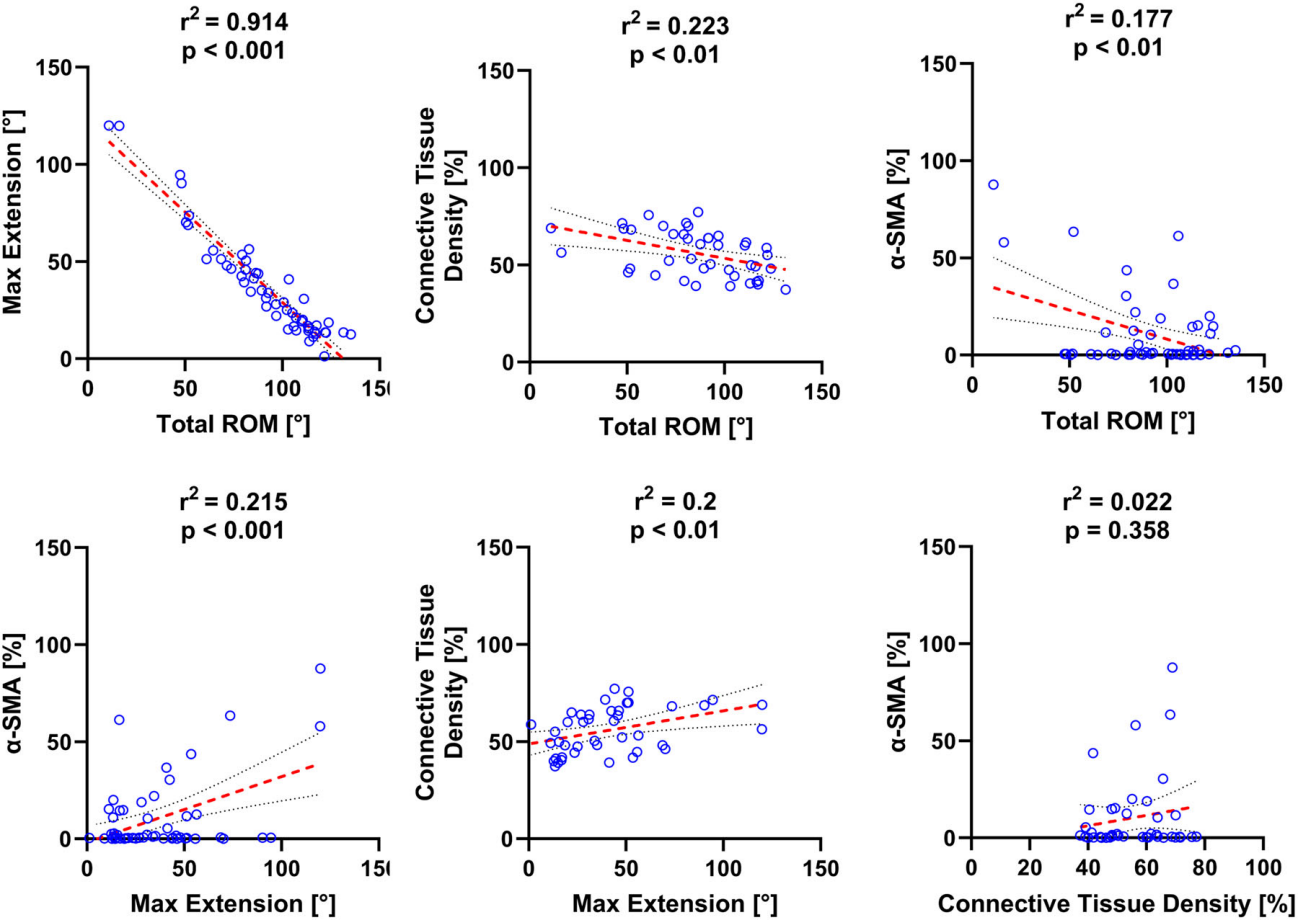
Linear regressions between each of the outcome measurements (r^2^ – coefficient of determination, p – significance). Best‐fit lines are denoted with red dashes. The black dotted lines represent the 95% confidence interval of the best‐fit line.

An examination of the relationships between the four main outcome variables, examined via linear regression analysis (Figure 12, Supporting Information S1: Table S3), revealed a strong correlation (r^2^ = 0.914, *p* < 0.001) between maximum extension angle and total ROM. No correlation was found between the connective tissue density and the percentage of α‐SMA‐positive cells. Weak correlations were observed between all other outcome measurements.

## 4 DISCUSSION

Our data from mechanical testing were consistent with Lake et al.^34^ and indicate that the Long Evans rat elbow contracture model is reproducible. Overall, capsule release surgery improved maximum extension, ROM, and connective tissue density. However, the improvements observed after 1 week were reduced at 3‐weeks postsurgery, indicating that the contracture may return following surgery. This reversal appeared in both our mechanical data and histological analysis. The return of contracture is often observed clinically in patients that undergo capsular release surgery, which can be frustrating for both patient and surgeon. Although the improvements were not statistically significant, the fact that our model reflects the clinical situation is an encouraging development that could help us better understand what is responsible for the contracture and how this surgical procedure can impact the process of healing. Further, it could help us evaluate the effectiveness of various therapeutic strategies.

By itself, remobilization of the injured arm was not enough to improve mechanical outcomes, similar to what was observed in a previous study using this model.^35^ We noticed that variability in the healing response was quite large after 3 weeks of remobilization, and that this variability diminished considerably after an additional 3 weeks of remobilization. We speculate that 3 weeks of remobilization was not enough time to realize mechanical improvements for all animals, but 6 weeks was enough, hence the drop in variability.

Interestingly, the connective tissue density seen in the *6W Remobile* group was similar to that of the *Uninjured* group. A return to a comparable ROM and maximum extension, however, was not reflected in the mechanical data for *6W Remobile*. We speculate that 12 weeks after injury (*6W Remobile*) is enough time for some inflammation and fibrosis to resolve such that the rest of the capsule returns to normal while the anterior portion remains fibrotic. Such a response would result in restricted joint movement and create a disconnect between the mechanical and histological outcomes we observed. In contrast, 6 weeks (*Injury Only*) and 9 weeks (*3W Remobile*) after the initial surgical injury is not enough time for inflammation and fibrosis in the joint capsule to subside. Similarly, although capsule release surgery ultimately improved joint biomechanics compared to the *3W* and *6W Remobile* groups, the surgery itself acted as an injury that sustained the inflammatory response. This renewed inflammatory response may be responsible for the return of contracture observed in our model and in the clinic.

In this study, the addition of daily blebbistatin injections appeared to reduce the connective tissue density, indicating that the drug may be mitigating the return of contracture. The reduction in connective tissue density following 3 weeks of blebbistatin injections is similar to what was found 1 week after capsule release surgery (*CRS* + *1W Recovery*) and 3 weeks of remobilization (*3W Remobile*). However, these histological changes again were not observed in our mechanical data (ROM and max extension). One explanation might be that blebbistatin had limited access to the fibrous tissue on the anterior aspect of the joint at sufficient concentrations to have an effect mechanically.

The lack of effect from blebbistatin treatment could also be because the drug was cleared from the circulation faster than therapeutic amounts were able to influence the anterior joint capsule. Previous in vitro research has found that blebbistatin has a half‐life of 20.2 min in rat hepatocytes.^44^ *In vivo*, blebbistatin was found to accumulate in the skeletal muscle of rats over the course of 45 min to about 15 μM after a 1 mg (~3.6 mg/kg) intraperitoneal dose of blebbistatin in DMSO. Blebbistatin concentration decreased below 5 μM in blood, cardiac muscle, brain, kidney, lung, and liver samples in the same rats.^44^ The amount of blebbistatin that was present in the joint was not measured in this study. Earlier work that examined the biodistribution of another hydrophobic small molecule, sulfasalazine, found that its concentration in synovial fluid is similar to its plasma concentration with an average synovial fluid‐to‐plasma ratio of 1.07 μg/mL.^45^ If blebbistatin distributes in a similar fashion as sulfasalazine, we could assume that concentrations found in the synovial fluid would be under 5 μM, well below the 50 μM used to reduce fibrin gel compaction and force generation in our previous in vitro studies.^32^, ^33^ Moreover, subcutaneous injections of small molecules have been shown to have decreased systemic exposure compared to intraperitoneal injections.^46^ Thus, the switch to subcutaneous injection, made to reduce the chance of aggravating the elbow, may have further limited blebbistatin's efficacy.

If further work is to be done using blebbistatin, it may need to be delivered locally since, due to limited solubility, there is little room to increase the amount of drug delivered systemically. Our previous work using a rabbit model of knee arthrofibrosis found that sulfasalazine, delivered locally, reduced knee stiffness.^47^ From this, we know that arthrofibrosis can be treated pharmacologically and that local drug delivery is a viable option. We are also exploring the use of lipid nanoparticles as a localized, injectable drug delivery system for hydrophobic molecules, such as blebbistatin and sulfasalazine. This strategy could potentially overcome issues regarding low or unsustained drug concentration in the joint capsule.

Other possible reasons for the lack of effect from blebbistatin treatment could be that cells other than myofibroblasts are responsible for contracture or that myofibroblasts are only temporarily present in the joint space. While myofibroblasts are often found in fibrotic joints, their presence and persistence do not appear to be universal for all joints and injuries. For example, some studies have reported an absence of myofibroblasts in frozen shoulders,^48^, ^49^ potentially because the patients were in the early inflammatory stage of the disease. In other studies, myofibroblasts were only present in the early stages of contracture in a rabbit knee model^19^ and the human elbow.^50^ In contrast, Hildebrand et al. reported that myofibroblasts were abundant in both early^21^ and chronic stages of posttraumatic joint contracture in both humans and rabbits.^17^, ^20^

Our results found no correlation between the connective tissue density and the percentage of α‐SMA‐positive cells and only weak correlations between the mechanical data and α‐SMA‐positive cells, which supports the idea that myofibroblasts may not be responsible for contracture, at least in the Long Evans Rat elbow model of contracture. More work needs to be done to clarify if and when myofibroblasts are present in the elbow joint capsule following trauma to better inform when adjunctive treatments should be applied.

Only 4 out of the 27 injured arms had percentages of α‐SMA‐positive cells greater than 30%. Most had percentages lower than 20%. There was also only one significant difference between groups (*Uninjured* vs. *3W Remobile*). The apparent disconnect α‐SMA has with connective tissue density and mechanical outcomes might be due to differences in how fibrotic tissue forms. Usher et al. suggests that there are two types of arthrofibrosis, active and residual.^51^ Active arthrofibrosis describes a condition where inflammatory processes and ECM deposition are continuous and driven by positive feedback loops. Patients with active fibrosis are those that experience pain and swelling in addition to reduced ROM. Residual arthrofibrosis describes joints with reduced ROM, but the inflammatory phase has resolved. In our study, the elbows that exhibited reduced mechanical parameters without the corresponding increase in α‐SMA‐positive cells may be examples of residual arthrofibrosis. Elbows that had increased percentages of α‐SMA‐positive cells may be examples of active arthrofibrosis. Future studies that examine inflammatory cytokines are needed to determine if this is the case.

A previous study using this model found that male and female rats developed similar deficits in mechanical outcomes.^52^ Therefore, only male rats were used in this study to reduce sample numbers and cost. It is possible, however, that sex‐dependent differences could emerge from using different treatment strategies, such as blebbistatin. We hope to address this limitation in future work by testing both male and female rats.

## 5 CONCLUSION

Capsule release surgery improved elbow range of motion in a rat model of posttraumatic joint contracture. However, the improvements seen 1 week after capsule release were reduced after 3 weeks, despite the addition of adjuvant treatment. The addition of blebbistatin treatments appeared to reduce fibrotic tissues in the joint, but these changes did not coincide with better mechanical outcomes. Due to the small sample size, however, these results should be considered preliminary. Future work will investigate relationships between treatment, fibrotic tissue deposition, myofibroblast activity, and biomechanics to determine if blebbistatin (or another compound) is a useful adjunctive therapy together with surgical intervention for treating joint contracture.

## AUTHOR CONTRIBUTIONS

**Austin J. Scholp:** Experimental design; data collection; data analysis; manuscript writing/editing. **Jordan A. Jensen:** Experimental design; data collection; manuscript writing/editing. **Timothy P. Fowler:** Experimental design; manuscript writing/editing. **Emily Petersen:** Experimental design; manuscript writing/editing. **Douglas Fredericks:** Experimental design; manuscript writing/editing. **Aliasger K. Salem:** Experimental design; manuscript writing/editing. **Dongrim Seol:** Data collection; data analysis; manuscript writing/editing. **Mitchell Coleman:** Data collection; manuscript writing/editing. **Spencer P. Lake:** Manuscript writing/editing. **James A. Martin:** Experimental design; data analysis; manuscript writing/editing. **Edward A. Sander:** Experimental design; data analysis; manuscript writing/editing. All authors have read and approved the final submitted manuscript.

## CONFLICT OF INTEREST STATEMENT

The authors declare no conflict of interest.

## Supporting information


**Figure S1.** Method to calculate connective tissue density. (A) A region of interest (ROI) was drawn in blue. (B) The image was converted to grayscale. (C) Thresholding was applied to obtain black and white images where white pixels represent connective tissue. The number of white pixels divided by the total number of pixels was used to obtain connective tissue density.

**Figure S2.** Method for training an AI model to automatically count cells that are either positive or negative for α‐SMA. (A) From the full scan of the immunolabeled slide, a 5x magnification image of the anterior aspect of the elbow joint was captured. (B) The image was trimmed of bone and muscle tissue. (C) The trimmed image was split into subsections. (D) Negative cells (Blue) were manually marked in the outlined subsection. (E) α‐SMA‐positive cells (yellow) were manually marked within the outlined subsection. Supporting information.

Supporting information.

## References

[1] Kuhn MA , Ross G . Acute elbow dislocations. Orthop Clin North Am. 2008;39(2):155‐161. 18374806 10.1016/j.ocl.2007.12.004

[2] Mehlhoff TL , Noble PC , Bennett JB , Tullos HS . Simple dislocation of the elbow in the adult. Results after closed treatment. J Bone Joint Surg Am. 1988;70(2):244‐249. 3343270

[3] Wessel LE , Gu A , Richardson SS , Fufa DT , Osei DA . Elbow contracture following operative fixation of fractures about the elbow. JSES Open Access. 2019;3(4):261‐265. 31891023 10.1016/j.jses.2019.09.004 PMC6928310

[4] Doornberg JN , Van Duijn PJ , Linzel D , et al. Surgical treatment of intra‐articular fractures of the distal part of the humerus: functional outcome after twelve to thirty years. J Bone Joint Surg Am. 2007;89(7):1524‐1532. 17606792 10.2106/JBJS.F.00369

[5] Myden C , Hildebrand K . Elbow joint contracture after traumatic injury. J Shoulder Elbow Surg. 2011;20(1):39‐44. 21050779 10.1016/j.jse.2010.07.013

[6] Tang C , Roidis N , Itamura J , Vaishnau S , Shean C , Stevanovic M . The effect of simulated elbow arthrodesis on the ability to perform activities of daily living. J Hand Surg [Am]. 2001;26(6):1146‐1150. 10.1053/jhsu.2001.28940 11721267

[7] Sotereanos DG , Darlis NA , Wright TW , Goitz RJ , King GJ . Unstable fracture‐dislocations of the elbow. Instr Course Lect. 2007;56:369‐376. 17472320

[8] Chan K , King GJW , Faber KJ . Treatment of complex elbow fracture‐dislocations. Curr Rev Musculoskelet Med. 2016;9:185‐189. 26984334 10.1007/s12178-016-9337-8 PMC4896880

[9] Higgs ZCJ , Danks BA , Sibinski M , Rymaszewski LA . Outcomes of open arthrolysis of the elbow without post‐operative passive stretching. J Bone Joint Surg Br. 2012;94(3):348‐352. 22371542 10.1302/0301-620X.94B3.27278

[10] Schrumpf MA , Lyman S , Do H , et al. Incidence of postoperative elbow contracture release in New York State. J Hand Surg [Am]. 2013;38(9):1746‐1752.e3. 10.1016/j.jhsa.2013.05.005 23831364

[11] Kulkarni GS , Kulkarni VS , Shyam AK , Kulkarni RM , Kulkarni MG , Nayak P . Management of severe extra‐articular contracture of the elbow by open arthrolysis and a monolateral hinged external fixator. J Bone Joint Surg Br. 2010;92(1):92‐97. 20044685 10.1302/0301-620X.92B1.22241

[12] Lindenhovius ALC , van de Luijtgaarden K , Ring D , Jupiter J . Open elbow contracture release: postoperative management with and without continuous passive motion. J Hand Surg [Am]. 2009;34(5):858‐865. 10.1016/j.jhsa.2009.01.003 19362791

[13] Charalambous CP , Morrey BF . Posttraumatic elbow stiffness. J Bone Jt Surg. 2012;94(15):1428‐1437. 10.2106/JBJS.K.00711 22854997

[14] Barlow JD , Morrey ME , Hartzler RU , et al. Effectiveness of rosiglitazone in reducing flexion contracture in a rabbit model of arthrofibrosis with surgical capsular release: a biomechanical, histological, and genetic analysis. Bone Joint Res. 2016;5(1):11‐17. 26813567 10.1302/2046-3758.51.2000593 PMC5009236

[15] Ring D , King G . Radial head arthroplasty with a modular metal spacer to treat acute traumatic elbow instability: surgical technique. J Bone Jt Surg. 2008;90(suppl ment_2_Part_1):63‐73. 10.2106/JBJS.G.01248 18310687

[16] Germscheid NM , Hildebrand KA . Regional variation is present in elbow capsules after injury. Clin Orthop Relat Res(1976–2007). 2006;450:219‐224. PMC2970598 17001766

[17] Hildebrand KA , Zhang M , van Snellenberg W , King GJW , Hart DA . Myofibroblast numbers are elevated in human elbow capsules after trauma. Clin Orthop Relat Res. 2004;419:189‐197. 10.1097/00003086-200402000-00031 PMC2950171 15021153

[18] Hildebrand KA , Zhang M , Hart DA . Myofibroblast upregulators are elevated in joint capsules in posttraumatic contractures. Clin Orthop Relat Res. 2007;456:85‐91. 17195814 10.1097/BLO.0b013e3180312c01 PMC2970597

[19] Abdel MP , Morrey ME , Barlow JD , et al. Myofibroblast cells are preferentially expressed early in a rabbit model of joint contracture. J Orthop Res. 2012;30(5):713‐719. 22057979 10.1002/jor.21588

[20] Hildebrand KA , Sutherland C , Zhang M . Rabbit knee model of post‐traumatic joint contractures: the long‐term natural history of motion loss and myofibroblasts. J Orthop Res. 2004;22(2):313‐320. 15013090 10.1016/j.orthres.2003.08.012

[21] Hildebrand KA , Zhang M , Germscheid NM , Wang C , Hart DA . Cellular, matrix, and growth factor components of the joint capsule are modified early in the process of posttraumatic contracture formation in a rabbit model. Acta Orthop. 2008;79(1):116‐125. 18283583 10.1080/17453670710014860 PMC2950862

[22] Hinz B . Myofibroblasts. Exp Eye Res. 2016;142:56‐70. 26192991 10.1016/j.exer.2015.07.009

[23] De Jesus AM , Aghvami M , Sander EA . A combined in vitro imaging and multi‐scale modeling system for studying the role of cell matrix interactions in cutaneous wound healing. PLoS One. 2016;11(2):e0148254. 26840835 10.1371/journal.pone.0148254 PMC4739727

[24] Hildebrand KA . Posttraumatic elbow joint contractures: defining pathologic capsular mechanisms and potential future treatment paradigms. J Hand Surg [Am]. 2013;38(11):2227‐2233. 10.1016/j.jhsa.2013.07.031 24075128

[25] Li B , Wang JHC . Application of sensing techniques to cellular force measurement. Sensors. 2010;10(11):9948‐9962. 22163449 10.3390/s101109948 PMC3231038

[26] Southern BD , Grove LM , Rahaman SO , et al. Matrix‐driven myosin II mediates the pro‐fibrotic fibroblast phenotype. J Biol Chem. 2016;291(12):6083‐6095. 26763235 10.1074/jbc.M115.712380 PMC4813589

[27] Bond JE , Ho TQ , Selim MA , Hunter CL , Bowers EV , Levinson H . Temporal spatial expression and function of non‐muscle myosin II isoforms IIA and IIB in scar remodeling. Lab Invest. 2011;91(4):499‐508. 21102503 10.1038/labinvest.2010.181 PMC3407540

[28] Even‐Ram S , Doyle AD , Conti MA , Matsumoto K , Adelstein RS , Yamada KM . Myosin IIA regulates cell motility and actomyosin–microtubule crosstalk. Nature Cell Biol. 2007;9(3):299‐309. 17310241 10.1038/ncb1540

[29] Vicente‐Manzanares M , Ma X , Adelstein RS , Horwitz AR . Non‐muscle myosin II takes centre stage in cell adhesion and migration. Nat Rev Mol Cell Biol. 2009;10(11):778‐790. 19851336 10.1038/nrm2786 PMC2834236

[30] Straight AF , Cheung A , Limouze J , et al. Dissecting temporal and spatial control of cytokinesis with a myosin II inhibitor. Science. 2003;299(5613):1743‐1747. 12637748 10.1126/science.1081412

[31] Kovács M , Tóth J , Hetényi C , Málnási‐Csizmadia A , Sellers JR . Mechanism of blebbistatin inhibition of myosin II. J Biol Chem. 2004;279(34):35557‐35563. 15205456 10.1074/jbc.M405319200

[32] Scholp AJ , Jensen J , Chinnathambi S , et al. Force‐bioreactor for assessing pharmacological therapies for mechanobiological targets. Front Bioeng Biotechnol. 2022;10:907611. 35928948 10.3389/fbioe.2022.907611 PMC9343955

[33] Atluri K , De Jesus AM , Chinnathambi S , et al. Blebbistatin‐loaded poly (d, l‐lactide‐co‐glycolide) particles for treating arthrofibrosis. ACS Biomater Sci Eng. 2016;2(7):1097‐1107. 33445238 10.1021/acsbiomaterials.6b00082

[34] Lake SP , Castile RM , Borinsky S , Dunham CL , Havlioglu N , Galatz LM . Development and use of an animal model to study post‐traumatic stiffness and contracture of the elbow. J Orthop Res. 2016;34(2):354‐364. 26177969 10.1002/jor.22981

[35] Dunham CL , Castile RM , Havlioglu N , Chamberlain AM , Lake SP . Temporal patterns of motion in flexion‐extension and pronation‐supination in a rat model of posttraumatic elbow contracture. Clin Orthop Relat Res. 2018;476(9):1878‐1889. 30001292 10.1097/CORR.0000000000000388 PMC6259801

[36] Dunham CL , Castile RM , Havlioglu N , Chamberlain AM , Galatz LM , Lake SP . Persistent motion loss after free joint mobilization in a rat model of post‐traumatic elbow contracture. J Shoulder Elbow Surg. 2017;26(4):611‐618. 28081997 10.1016/j.jse.2016.09.059 PMC5502529

[37] Whishaw IQ , Gorny B , Foroud A , Kleim JA . Long–Evans and Sprague–Dawley rats have similar skilled reaching success and limb representations in motor cortex but different movements: some cautionary insights into the selection of rat strains for neurobiological motor research. Behav Brain Res. 2003;145(1‐2):221‐232. 14529819 10.1016/s0166-4328(03)00143-8

[38] Sacrey LAR , Alaverdashvili M , Whishaw IQ . Similar hand shaping in reaching‐for‐food (skilled reaching) in rats and humans provides evidence of homology in release, collection, and manipulation movements. Behav Brain Res. 2009;204(1):153‐161. 19520119 10.1016/j.bbr.2009.05.035

[39] Aoki S , Sato Y , Yanagihara D . Characteristics of leading forelimb movements for obstacle avoidance during locomotion in rats. Neurosci Res. 2012;74(2):129‐137. 22902354 10.1016/j.neures.2012.07.007

[40] Durk MR , Deshmukh G , Valle N , Ding X , Liederer BM , Liu X . Use of subcutaneous and intraperitoneal administration methods to facilitate cassette dosing in microdialysis studies in rats. Drug Metab Dispos. 2018;46(7):964‐969. 29700231 10.1124/dmd.118.080697

[41] Owen AR , Dagneaux L , Limberg AK , et al. Biomechanical, histological, and molecular characterization of a new posttraumatic model of arthrofibrosis in rats. J Orthop Res. 2022;40(2):323‐337. 33871082 10.1002/jor.25054 PMC8523596

[42] Hinz B . The myofibroblast: paradigm for a mechanically active cell. J Biomech. 2010;43(1):146‐155. 19800625 10.1016/j.jbiomech.2009.09.020

[43] Temporin K , Shimada K , Oura K , Owaki H . Arthroscopic release for the severely stiff elbow. Musculoskelet Surg. 2020;104:81‐86. 30945150 10.1007/s12306-019-00601-6

[44] Gyimesi M , Rauscher AÁ , Suthar SK , Rauscher AÁ , Suthar SK , et al. Improved inhibitory and absorption, distribution, metabolism, excretion, and toxicology (ADMET) properties of blebbistatin derivatives indicate that blebbistatin scaffold is ideal for drug development targeting myosin‐2. J Pharmacol Exp Ther. 2021;376(3):358‐373. 33468641 10.1124/jpet.120.000167

[45] Farr M , Brodrick A , Bacon PA . Plasma and synovial fluid concentrations of sulphasalazine and two of its metabolites in rheumatoid arthritis. Rheumatol Int. 1985;5:247‐251. 2906452 10.1007/BF00541351

[46] Al Shoyaib A , Archie SR , Karamyan VT . Intraperitoneal route of drug administration: should it be used in experimental animal studies. Pharm Res. 2020;37:12. 10.1007/s11095-019-2745-x PMC7412579 31873819

[47] Atluri K , Brouillette MJ , Seol D , et al. Sulfasalazine resolves joint stiffness in a rabbit model of arthrofibrosis. J Orthop Res. 2020;38(3):629‐638. 31692083 10.1002/jor.24499

[48] Hand GCR , Athanasou NA , Matthews T , Carr AJ . The pathology of frozen shoulder. J Bone Joint Surg Br. 2007;89(7):928‐932. 17673588 10.1302/0301-620X.89B7.19097

[49] Bunker T , Anthony P . The pathology of frozen shoulder. A Dupuytren‐like disease. J Bone Joint Surg Br. 1995;77(5):677‐683. 7559688

[50] Doornberg JN , Bosse T , Cohen MS , Jupiter JB , Ring D , Kloen P . Temporary presence of myofibroblasts in human elbow capsule after trauma. J Bone Jt Surg. 2014;96(5):e36. 10.2106/JBJS.M.00388 24599208

[51] Usher KM , Zhu S , Mavropalias G , Carrino JA , Zhao J , Xu J . Pathological mechanisms and therapeutic outlooks for arthrofibrosis. Bone Res. 2019;7(1):9. 30937213 10.1038/s41413-019-0047-x PMC6433953

[52] Reiter AJ , Schott HR , Castile RM , et al. Females and males exhibit similar functional, mechanical, and morphological outcomes in a rat model of posttraumatic elbow contracture. J Orthop Res. 2021;39(9):2062‐2072. 33222267 10.1002/jor.24918 PMC8140065

