# Supplementary figures and images for: Capsule release surgery temporarily reduces contracture in a rat elbow model of arthrofibrosis
Source: J Orthop Res. 2024 Sep 15;43(1):23–36. doi: 10.1002/jor.25967 (PMC11615420; doi:10.1002/jor.25967)

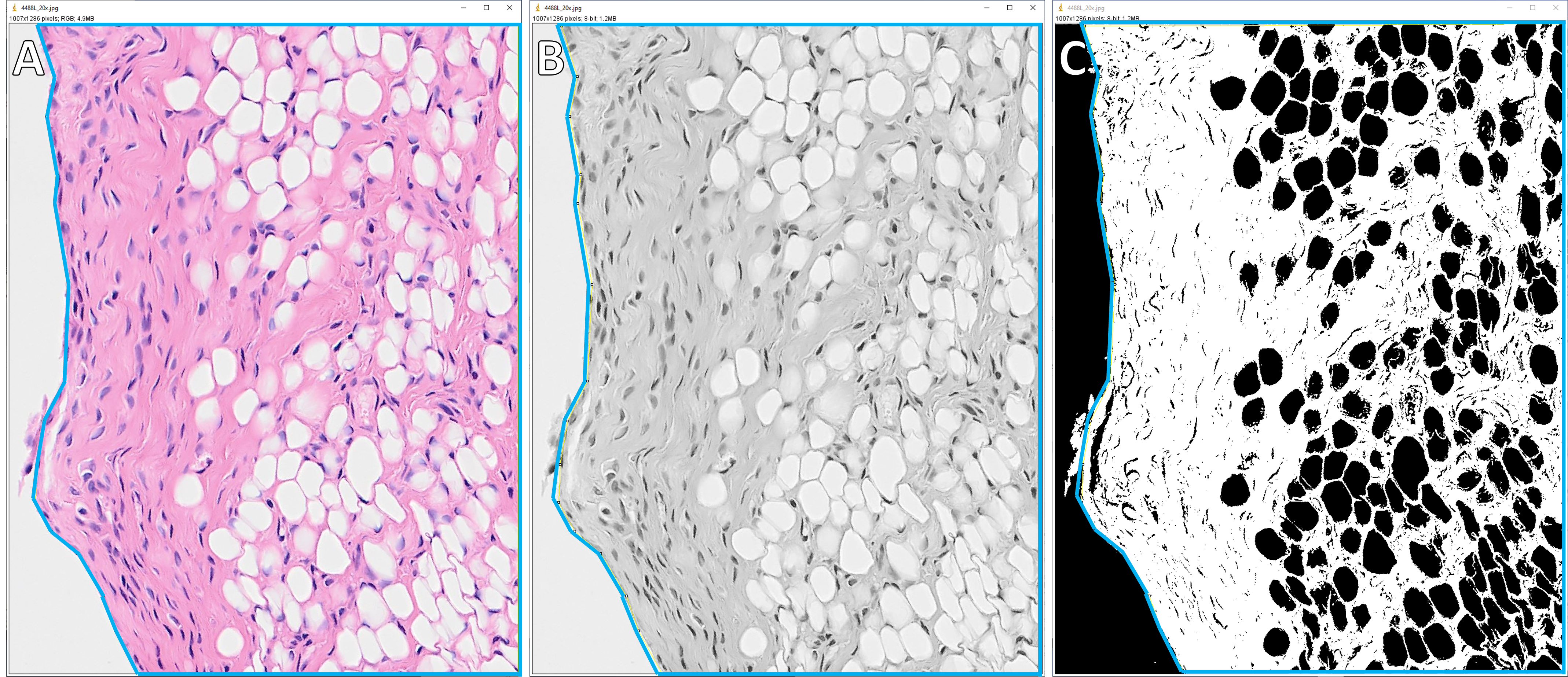

Supplement: Supplementary file 1 — Figure S1. Method to calculate connective tissue density. (A) A region of interest (ROI) was drawn in blue. (B) The image was converted to grayscale. (C) Thresholding was applied to obtain black and white images where white pixels represent connective tissue. The number of white pixels divided by the total number of pixels was used to obtain connective tissue density. [file JOR-43-23-s002.tif]

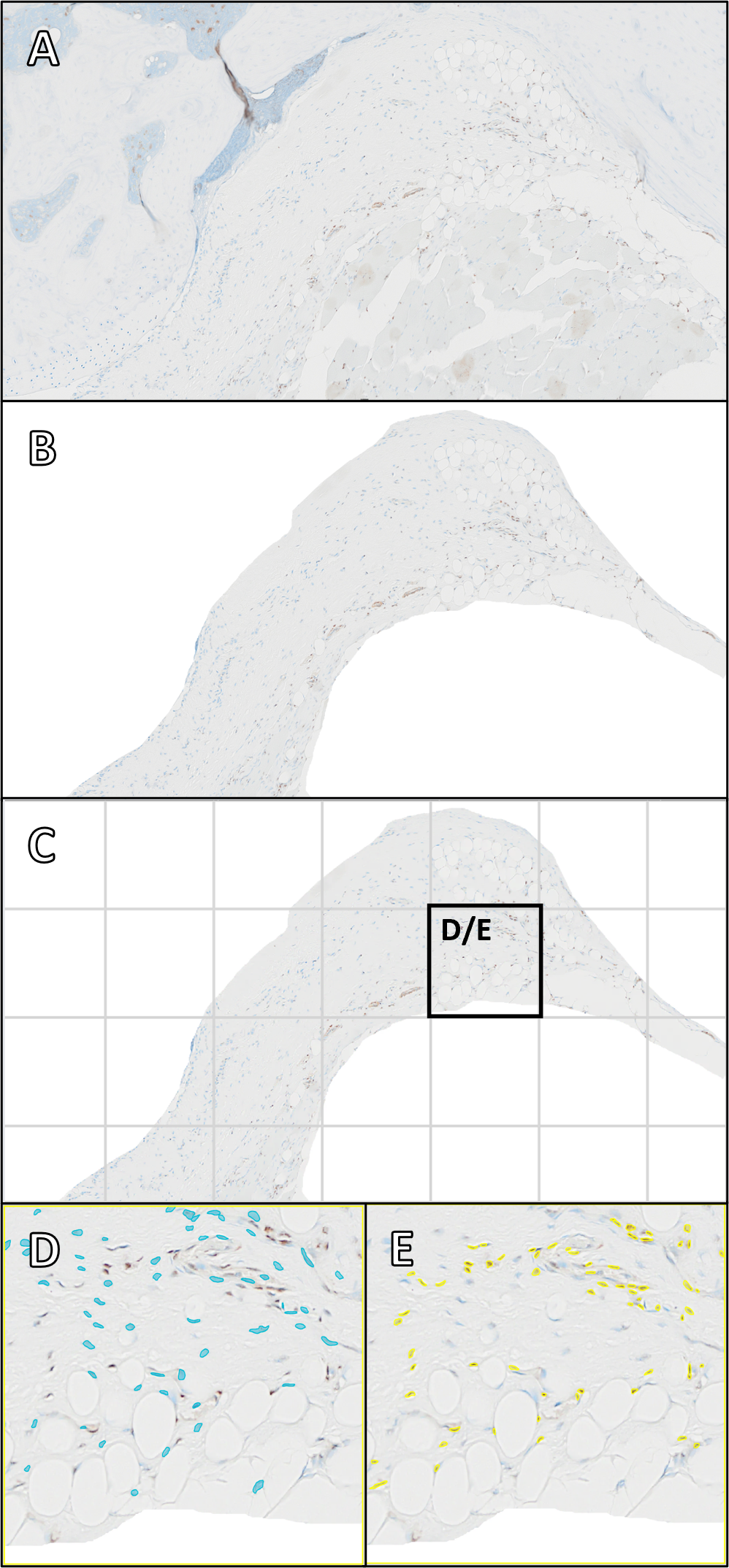

Supplement: Supplementary file 2 — Figure S2. Method for training an AI model to automatically count cells that are either positive or negative for α‐SMA. (A) From the full scan of the immunolabeled slide, a 5x magnification image of the anterior aspect of the elbow joint was captured. (B) The image was trimmed of bone and muscle tissue. (C) The trimmed image was split into subsections. (D) Negative cells (Blue) were manually marked in the outlined subsection. (E) α‐SMA‐positive cells (yellow) were manually marked within the outlined subsection. Supporting information. [file JOR-43-23-s003.tif]
