# Supplementary material for: Capsule release surgery temporarily reduces contracture in a rat elbow model of arthrofibrosis
Source: J Orthop Res. 2024 Sep 15;43(1):23–36. doi: 10.1002/jor.25967 (PMC11615420; doi:10.1002/jor.25967)
Supplement: Supplementary file 3 — Supporting information. [file JOR-43-23-s001.docx]

**Table S1.** Results from individual comparisons between all groups for range of motion (ROM) and maximum extension angles. Significant differences are marked in bold.

|  |  | **p-values** | |
| --- | --- | --- | --- |
| **Group A** | **Group B** | **ROM** | **Max Extension** |
| Uninjured | Injury Only | **<.001** | **<.001** |
|  | 3W Remobile | **<.001** | **<.001** |
|  | 6W Remobile | **<.001** | **<.001** |
|  | Surgery + 1W Recovery | 0.054 | 0.240 |
|  | Surgery + 3W Recovery | **0.002** | **<.001** |
|  | Surgery + 3W Daily Vehicle | **0.006** | **0.010** |
|  | Surgery + 3W Daily Blebb | **0.001** | **0.025** |
| Injury Only | 3W Remobile | 0.129 | 0.175 |
|  | 6W Remobile | 0.168 | **0.009** |
|  | Surgery + 1W Recovery | **<.001** | **<.001** |
|  | Surgery + 3W Recovery | **0.003** | **0.001** |
|  | Surgery + 3W Daily Vehicle | **0.001** | **<.001** |
|  | Surgery + 3W Daily Blebb | **0.004** | **<.001** |
| 3W Remobile | 6W Remobile | >.999 | 0.897 |
|  | Surgery + 1W Recovery | 0.257 | **0.008** |
|  | Surgery + 3W Recovery | 0.800 | 0.495 |
|  | Surgery + 3W Daily Vehicle | 0.612 | **0.103** |
|  | Surgery + 3W Daily Blebb | 0.859 | **0.058** |
| 6W Remobile | Surgery + 1W Recovery | 0.199 | 0.196 |
|  | Surgery + 3W Recovery | 0.722 | 0.996 |
|  | Surgery + 3W Daily Vehicle | 0.524 | 0.763 |
|  | Surgery + 3W Daily Blebb | 0.792 | 0.611 |
| Surgery + 1W Recovery | Surgery + 3W Recovery | 0.984 | 0.586 |
|  | Surgery + 3W Daily Vehicle | 0.999 | 0.974 |
|  | Surgery + 3W Daily Blebb | 0.967 | 0.995 |
| Surgery + 3W Recovery | Surgery + 3W Daily Vehicle | >.999 | 0.987 |
|  | Surgery + 3W Daily Blebb | >.999 | 0.951 |
| Surgery + Daily Vehicle | Surgery + 3W Daily Blebb | >.999 | >.999 |

**Table S2.** Results for individual comparisons between groups for connective tissue density. Significant p-values are marked in bold.

| **Group A** | **Group B** | **p-value** |
| --- | --- | --- |
| Uninjured | Injury Only | **<.001** |
|  | 3W Remobile | **0.002** |
|  | 6W Remobile | >.999 |
|  | Surgery + 1W Recovery | **0.002** |
|  | Surgery + 3W Recovery | **<.001** |
|  | Surgery + 3W Daily Vehicle | **<.001** |
|  | Surgery + 3W Daily Blebb | **<.001** |
| Injury Only | 3W Remobile | 0.406 |
|  | 6W Remobile | **<.001** |
|  | Surgery + 1W Recovery | 0.43 |
|  | Surgery + 3W Recovery | >.999 |
|  | Surgery + 3W Daily Vehicle | >.999 |
|  | Surgery + 3W Daily Blebb | 0.612 |
| 3W Remobile | 6W Remobile | 0.073 |
|  | Surgery + 1W Recovery | >.999 |
|  | Surgery + 3W Recovery | 0.593 |
|  | Surgery + 3W Daily Vehicle | 0.299 |
|  | Surgery + 3W Daily Blebb | >.999 |
| 6W Remobile | Surgery + 1W Recovery | 0.066 |
|  | Surgery + 3W Recovery | **<.001** |
|  | Surgery + 3W Daily Vehicle | **<.001** |
|  | Surgery + 3W Daily Blebb | **0.032** |
| Surgery + 1W Recovery | Surgery + 3W Recovery | 0.621 |
|  | Surgery + 3W Daily Vehicle | 0.321 |
|  | Surgery + 3W Daily Blebb | >.999 |
| Surgery + 3W Recovery | Surgery + 3W Daily Vehicle | >.999 |
|  | Surgery + 3W Daily Blebb | 0.805 |
| Surgery + Daily Vehicle | Surgery + 3W Daily Blebb | 0.502 |

**Table S3.** R-squared and p-values for correlations between each outcome measurement. Significant p-values are marked in bold.

| **Group A** | **Group B** | **p-value** | **R-squared** |
| --- | --- | --- | --- |
| Total ROM | Max Extension | **<.001** | 0.914 |
|  | Connective Tissue Density | **0.002** | 0.223 |
|  | Percent α-SMA | **0.002** | 0.177 |
| Max Extension | Connective Tissue Density | **0.003** | 0.200 |
|  | Percent α-SMA | **<.001** | 0.215 |
| Connective Tissue Density | Percent α-SMA | 0.358 | 0.022 |
